# Mapping intra-urban malaria risk using high resolution satellite imagery: a case study of Dar es Salaam

**DOI:** 10.1186/s12942-016-0051-y

**Published:** 2016-07-30

**Authors:** Caroline W. Kabaria, Fabrizio Molteni, Renata Mandike, Frank Chacky, Abdisalan M. Noor, Robert W. Snow, Catherine Linard

**Affiliations:** 1 Spatial Health Metrics Group, Kenya Medical Research Institute/Wellcome Trust Research Programme, Nairobi, Kenya; 2 National Malaria Control Programme, Ministry of Health and Social Welfare, Dar es Salaam, Tanzania; 3 Swiss Tropical and Public Health Institute, Dar es Salaam, Tanzania; 4 Centre for Tropical Medicine and Global Health, Nuffield Department of Clinical Medicine, University of Oxford, Oxford, UK; 5 Department of Geography, Université de Namur, Rue de Bruxelles 61, 5000 Namur, Belgium; 6 Biological Control and Spatial Ecology, Université Libre de Bruxelles CP160/12, Av. F.D. Roosevelt 50, 1050 Brussels, Belgium

**Keywords:** Urban malaria, Remote sensing, Boosted Regression Trees, Dar es Salaam

## Abstract

**Background:**

With more than half of Africa’s population expected to live in urban settlements by 2030, the burden of malaria among urban populations in Africa continues to rise with an increasing number of people at risk of infection. However, malaria intervention across Africa remains focused on rural, highly endemic communities with far fewer strategic policy directions for the control of malaria in rapidly growing African urban settlements. The complex and heterogeneous nature of urban malaria requires a better understanding of the spatial and temporal patterns of urban malaria risk in order to design effective urban malaria control programs. In this study, we use remotely sensed variables and other environmental covariates to examine the predictability of intra-urban variations of malaria infection risk across the rapidly growing city of Dar es Salaam, Tanzania between 2006 and 2014.

**Methods:**

High resolution SPOT satellite imagery was used to identify urban environmental factors associated malaria prevalence in Dar es Salaam. Supervised classification with a random forest classifier was used to develop high resolution land cover classes that were combined with malaria parasite prevalence data to identify environmental factors that influence localized heterogeneity of malaria transmission and develop a high resolution predictive malaria risk map of Dar es Salaam.

**Results:**

Results indicate that the risk of malaria infection varied across the city. The risk of infection increased away from the city centre with lower parasite prevalence predicted in administrative units in the city centre compared to administrative units in the peri-urban suburbs. The variation in malaria risk within Dar es Salaam was shown to be influenced by varying environmental factors. Higher malaria risks were associated with proximity to dense vegetation, inland water and wet/swampy areas while lower risk of infection was predicted in densely built-up areas.

**Conclusions:**

The predictive maps produced can serve as valuable resources for municipal councils aiming to shrink the extents of malaria across cities, target resources for vector control or intensify mosquito and disease surveillance. The semi-automated modelling process developed can be replicated in other urban areas to identify factors that influence heterogeneity in malaria risk patterns and detect vulnerable zones. There is a definite need to expand research into the unique epidemiology of malaria transmission in urban areas for focal elimination and sustained control agendas.

**Electronic supplementary material:**

The online version of this article (doi:10.1186/s12942-016-0051-y) contains supplementary material, which is available to authorized users.

## Background

The rapid rate of urban growth in sub-Saharan Africa will mean that the majority of the population on the continent will be classified as urban by 2030 [1]. The process of urbanization is associated with changes in the demographic, environmental and socioeconomic landscapes which in turn impact on the health of urban residents [2–4], including their risks of vector-borne diseases [4–6].

Malaria in Africa has long been regarded as a rural disease with the process of urbanization reducing suitable breeding environments for the dominant vector species complexes of *Anopheles gambiae* s.l. and *A. funestus* [6–9]. However, the risk of malaria infection does persist within densely populated, urban settings of Africa. In particular, *A. gambiae* s.l. is more likely to be breed in urban aquatic habitats [10–12] than other vector species and has been found in domestic containers and highly organically polluted habitats in urban areas [13, 14]. The focal transmission risk in urban areas is associated with proximity to breeding sites due to within urban water bodies, urban agriculture and proximity to peri-urban peripheries more likely to support vector breeding [6, 8, 15–19]. This heterogeneity of intra-urban risk is not captured in continental malaria risk mapping initiatives [20–23] and are not considered as part of current national control strategies that focus protecting less densely populated rural communities where risk of infection is typically higher compared to neighbouring urban areas.

Remote Sensing and Geographical Information Systems (GIS) provide cost-effective tools to identify environmental risk factors for high risk areas of vector-borne diseases. Previous studies have showed that satellite-derived or ground defined mapped extents of water bodies, swampy areas and agricultural land use, can distinguish with some precision areas of higher malaria risk within the urban settings of Dar es Salaam, Tanzania [14, 16] Ouagadougou, Burkina Faso [24] and Dakar, Senegal [8, 25]. The studies in Dar es Salaam during the early 2000s used aerial photography and hand-drawn maps to digitize potential mosquito breeding sites which were compared to empirical measures of mosquito larval densities and limited data on the prevalence of malaria infection among school children [14, 16]. The authors were able to demonstrate spatial declines in school children’s infection prevalence from the periphery to the centre of Dar es Salaam [16] and higher larval densities were associated with closer proximity to urban agriculture [14]. High resolution satellite imagery are useful for accurate mapping of malaria risk factors in urban area and can be used to detect heterogeneity in land cover classes over small distances and improve the ability to identify urban vector breeding sites are often small, partially or completely covered by vegetation. High resolution imagery was shown to be most accurate in identifying *A. gambiae* larval habitats compared to lower resolution satellite imagery [26]. The main aim of this study was to identify at a high resolution environmental factors that influence localized heterogeneity of malaria transmission in the city of Dar es Salaam, Tanzania. In this study, we combine data derived from high resolution SPOT satellite image and a wider suite of remotely sensed variables to estimate their impact of intra-urban variations of malaria infection risk in Dar es Salaam between 2006 and 2014.

## Methods

### Study area

Dar es Salaam is Tanzania’s largest city with a population of 4.6 million people [27]. With an annual population growth rate of 5.6 %, Dar es Salaam is among the fastest growing cities in Africa and the metropolitan population of Dar es Salaam is projected to reach over 5 million by 2020. The city is located on the East African coast and has been endemic for *Plasmodium falciparum* transmission since the turn of the last century when it was occupied by German Colonial authorities [28]. *P. falciparum* accounts for over 90 % of cases treated within the city [16, 29] with over a million malaria cases reported annually by the health facilities in Dar es Salaam [29] although some of these cases could be imported cases. The commercial and administrative significance of the port city of Dar es Salaam, meant that it enjoyed a long history of aggressive control through mass drug administration under German control [16, 28], environmental management under British Colonial rule [30, 31] and since the 1970s periods of integrated vector management as part of municipality control efforts [16, 32] culminating in the current programme referred to as the Urban Malaria Control Project (UMCP) [15, 16, 29, 33, 34]. Largely community-based, the UMCP mainly focuses on integrated malaria vector control based on ground-based mapping and surveillance of potential mosquito breeding sites. Routine mosquito surveillance and larviciding is conducted by community-based resource persons (CORPs), recruited from local communities via the elected local government [29, 33, 35, 36]. However, ground-based mapping and surveillance has been reported as labour-intensive and expensive [15, 37]. The application of remote sensing as a faster and less labour intensive alternative for targeted and effective control application is explored in this study. In addition, we explore the use of parasite prevalence surveys in estimating urban malaria risk.

### Overview of analysis strategy

**Fig. 1:**
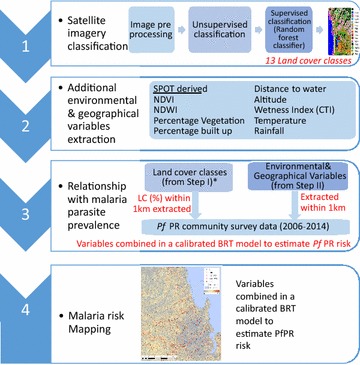
Conceptual framework used in the analysis of urban environmental factors that influence malaria prevalence in urban settings. *Note* the proportion of coverage of each LC class was extracted within a rectangular moving window of 1 km. Ancillary environmental variables assembled in Stage 2 were also extracted within a 1 km radius

Figure 1 gives an overview of the framework for analysis used in this study and described in more detail in subsequent sections.

### Satellite imagery classification

#### Image acquisition and pre-processing

High resolution satellite image from SPOT 6 (Système Pour l‘Observation de la Terre) at a resolution of 1.5 m, acquired during the short rains on 14th December 2012 was obtained for the city of Dar es Salaam. The satellite image includes four spectral bands blue (0.455–0.525 µm), green (0.530–0.590 µm), red (0.625–0.695 µm), and near-infrared (NIR) (0.760–0.890 µm). The image was geo-referenced and projected to UTM zones on the WGS84 datum. Atmospheric correction was then applied using a Dark Object Subtraction (DOS) model in image analysis software, ENVI version 5.0 (Exelis VlS, USA). Radiometric correction was conducted on the satellite image by first converting digital numbers to spectral radiance then calculating exoatmospheric reflectance (reflectance above the atmosphere) using published post-launch gain and offset values [38]. A coastline mask was digitized and applied to mask out pixels of the ocean from the subsequent analysis.

#### Unsupervised classification

The SPOT image was then classified to extract land cover (LC) classes. In the initial step of image classification, exploratory unsupervised classification was run to identify a manageable number of land cover classes for image training. Unsupervised classification using ISODATA algorithm repeated over 20 iterations was used to classify the satellite image into 20 LC classes. Class validation was conducted by checking accuracy of the generated classification for a random set of points in Google Earth. Several LC classes were merged in order to reduce the number of classes to 13, which were subsequently used to identify training sites that would be used for supervised classification in the second step of image classification. Similar methods of hybrid classification combining manual digitizing and semi-automated techniques to generate training sites for supervised classification have been used in previous studies [39–43].

#### Supervised classification

The output of the unsupervised classification was used to identify training classes for the supervised classification. For each of the 13 LC classes, a training dataset was selected on the satellite image by manually digitizing multiple training polygons for each class. One hundred training sites were obtained for each of the LC class.

A supervised classification algorithm based on random forest (RF) modelling was used to classify the satellite image [44–46]. Unlike the statistical algorithm used for the unsupervised classification that is based on the assumption that each cluster comes from a spherical normal distribution which is often not true for remote sensing images; the RF algorithm does not start with a predetermined model but instead learns the relationship from the data [45]. To build the RF model, spectral values were extracted from the multi-band SPOT image for each pixel within the training polygons. Optimal values for the number of trees (*N*) and number of observations per node (*m*) that maximize the classification accuracy while minimizing the computational time were selected by testing different combinations. Error rate estimates and confusion matrices were used to assess classification accuracy. All analyses were conducted using the statistical environment R (version 2.15.3). The RF model was developed using the random forest package version 4.6–7 [46] and additional functions provided in [47].

### Additional environmental and geographical variables extraction

Using the LC classes developed above, a vegetation predictor was determined by combining dense and riverine vegetation LC classes. Using focal statistics techniques, percentage vegetation was then calculated within a 1 km radius. Similarly, built-up classes were combined and percentage built-up pixel calculated within a 1 km radius. Euclidean distance tool in ArcGIS was used to calculate distance to water bodies for each pixel represented by the parasite prevalence survey location. The distance from inland water variable was calculated using water channels identified in the image combined with data from the Global Lakes and Wetlands Database (GLWD) to account for seasonal water channels in the study area that could have been present over the study period but not identified in 2012 satellite imagery.

In addition, several additional variables including humidity, vegetation and soil indicators were calculated from the SPOT image. The Normalized Difference Vegetation Index (NDVI) was calculated using the NIR and Red spectral bands as (NIR-Blue)/(NIR + Blue) while the Normalized Difference Water Index (NDWI) was calculated by normalising the difference between the green and NIR bands calculated as (NIR-Green)/(NIR + Green) [48]. The NDWI is useful in potentially delineating open water features while eliminating the presence of soil and terrestrial vegetation features. Ancillary environmental and geographical datasets shown to be associated with malaria transmission in the literature were acquired from secondary sources. Altitude was obtained from ASTER Digital Elevation Model (DEM) available at 30 m spatial resolution [49]. The Aster DEM was also used to calculate a Compound Topographic Index (CTI), a wetness index that is a function of topographic slope and the upstream contributing area orthogonal to the flow direction [50]. Mean monthly temperature for each month in the period 2006 to 2014 was calculated from land surface temperatures (LST) dataset extracted from daily Moderate Resolution Imaging Spectro-radiometer (MODIS-Terra) images. These would then be matched to the month of malaria prevalence survey for each community site. MODIS LST is freely available at 1 km spatial resolution [51]. Annual precipitation estimates in 2012 were calculated from daily rainfall estimates obtained from African Rainfall Estimates version 2 (RFE 2.0) dataset developed as a collaborative programme between NOAA’s Climate Prediction centre (CPC) and USAID/Famine Early Systems Network (FEWS) [52]. A summary of all covariates used in the model, their sources as well as their spatial resolution is given in the Table 1.

**Table 1:** Summary of variables used in BRT model to estimate *Pf*PR_2–10_ risk

Variable	Source	Data source spatial resolution
Land cover classes (C1–C13)	SPOT satellite image^a^	1.5 m
Percentage dense/riverine vegetation	SPOT satellite image^a^	1.5 m
Percentage built-up	SPOT satellite image^a^	1.5 m
Distance to inland water (m)	SPOT satellite image^a^ and GLWD^b^	1.5 m
		–
NDVI	SPOT satellite image^a^	1.5 m
NDWI	SPOT satellite image^a^	1.5 m
Altitude	ASTER GDEM^c^	30 m
Wetness Index (CTI)	ASTER GDEM^c^	30 m
Temperature	MODIS LST^d^	1 km
Rainfall	RFE 2.0^e^	1 km

All variables except distance to water were extracted within 1 km radius

*Data sources*: ^a^ SPOT imagery; complete section on SPOT data

^b^Global Lakes and Wetlands Database. www.worldwildlife.org/GLWD

^c^ http://www.jspacesystems.or.jp/ersdac/GDEM/E/index.html

^d^ https://lpdaac.usgs.gov/dataset_discovery/modis/modis_products_table/mod11a1

^e^ http://www.cpc.noaa.gov/products/international/data.shtml

### Relationship with malaria parasite prevalence: BRT modelling

In the third stage, community level parasite prevalence survey data was combined with LC classes (Stage 1) and other environmental factors (Stage 2) to identify environmental factors that influence malaria risk within the urban area.

#### *Plasmodium falciparum* parasite prevalence data

As part of continued support to the National Malaria Control Programme (NMCP) in Tanzania, the Information for Malaria Project (INFORM) has assembled from published and unpublished sources all available community based survey data on malaria infection prevalence for the country, including survey data from Dar es Salaam [53]. In brief, data included the month and year of the survey, numbers of individuals examined, lower and upper ages of the population sampled, methods used to detect parasites and the longitude and latitude of the community surveyed. Due to diversity in the age ranges of sampled populations between studies, there was need for a standardized age range to make meaningful comparisons of *Plasmodiumfalciparum* parasite rates across surveys. We therefore standardised parasite prevalence into the 2–10 years age group (*Pf*PR_2–10_) using algorithms based on catalytic conversion models first used in malaria by Pull and Grab [54] that uses the lower and upper range of the sample and the overall prevalence to transform into a predicted estimate in children aged 2–10 years as described in Smith et al. [55]. The working paper on data assembly under the INFORM project is available on the INFORM website (http://www.inform-malaria.org/working-papers/). Only data from 2006 to 2014 were selected for the analysis to coincide with periods of remotely sensed image used for urban risk classifications. The final dataset included 169 community surveys at 116 sample locations within Dar es Salaam. The majority of the data locations (*N* = 75, 65 %) were from surveys undertaken between 2011 and 2014, where 30 were undertaken as part of school based investigations of malaria risk undertaken by the NMCP in 2014. The majority (82 %) of the surveys were tested using RDT. A summary of key characteristics of the PR survey data is given in the Additional file 1.

The geographic coordinates of each community survey, measured at the estimated geographic centre of the survey site, were used as a unique identifier to extract values of the LC classes at the survey location. The extractions were done in ArcGIS 10 (ESRI, USA) using spatial neighbourhood analysis technique to obtain the proportion of coverage of each LC class within a rectangular moving window of 1 km radius surrounding each grid cell. Ancillary environmental variables assembled in Stage 2 were also extracted within a 1 km radius.

#### BRT modelling

Boosted Regression Tree (BRT) modelling was then used to examine the relationship between parasite prevalence (*Pf*PR_2–10_), urban LC classes as well as other environmental variables sampled at each community survey site. BRT is a machine learning technique increasingly used for modelling event distribution in ecology and epidemiology [56–59], in remote sensing land cover classification [60] and land cover change modelling [61].

To build the BRT model, the optimal number of trees *nt* was determined using the *gbm* function provided by Elith et al. [62]. Several combinations of the learning rate (LR) (0.025, 0.05, 0.1) and tree complexity (tc) (1, 5, 9) parameters were tested. Cross-validation techniques were used to evaluate model predictive performance, by randomly separating the dataset into a modelling dataset that was used to fit the model and a testing dataset that was excluded from model fitting and was used for testing the model’s predictive performance. The ratio model set was set at 75 % which defined the percentage of the data sampled at every run. This was further improved using bootstrapping techniques over 25 iterations. Root mean square error (RMSE) was used to select the optimal model using the smallest value. Important predictors of *Pf*PR_2–10_ were identified using the relative contribution output of the BRT model while the relationship patterns between individual predictors and *Pf*PR_2–10_ were examined through partial differential plots. Variables with zero influence or <1 % relative contribution were dropped from the model. BRT models were developed using the R package ‘gbm’ version 1.6–3.2 [63] and the additional functions provided in Elith et al. [62]. All analyses were conducted using R (version 2.15.3) [64].

### Malaria risk mapping

In the last stage, the final selected BRT model was used to predict malaria parasite prevalence on a 10 m grid level for the city of Dar es Salaam based on the identified associations between LC classes, environmental variables and parasite prevalence. To obtain more reliable results, the BRT model runs were repeated in 25 iterations with the mean predicted value over the 25 iterations calculated as the final value for each grid cell. The mean parasite prevalence was then estimated by ward, the lowest administrative unit level used for municipal planning in Dar es Salaam.

## Results

**Fig. 2:**
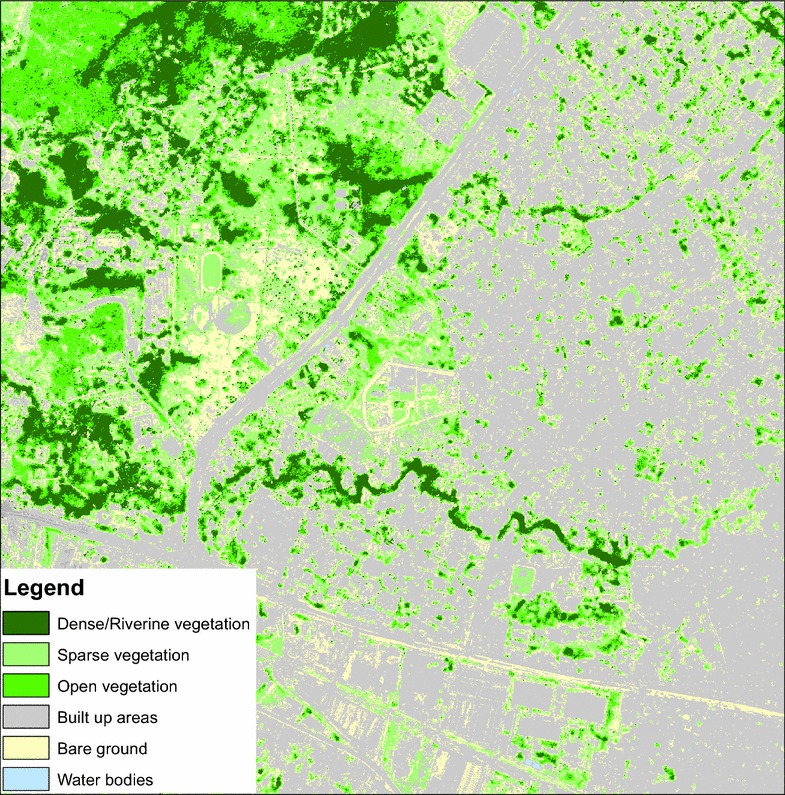
Thematic mapping showing the results of supervised classification of SPOT 6 satellite imagery using RF algorithm in section of Dar es Salaam city

The final classification defined 13 LC classes, distributed as five urban classes (depending on the type of buildings and tarmacked roads), three vegetation classes (light, dense or riverine), two water classes (inland or sea) and three bare soil classes (sand, bare soil, or bare soil mixed with vegetation) (Fig. 2). The accuracy of the image classification result was evaluated using an error matrix, one of the most widely used post-classification accuracy assessment methods. Overall classification accuracy of the satellite image covering Dar es Salaam was 87.1 % with Kappa co-efficient of 0.854.

Empirical estimates of malaria infection risk (*Pf*PR_2–10_) ranged from 0 to 38.8 %, with 18 % of surveys reporting zero infection among the 169 sample locations. The lowest RMSE value, indicative of the best fitting model, was used to determine the parameters of the final BRT model. Several combinations of the learning rate (LR) (0.025, 0.05, 0.1) and tree complexity (tc) (1, 5, 9) parameters were tested. The BRT model with the smallest RMSE value of 16.02 was selected as the most optimal model with the final tuning parameters set to: learning rate (LR) = 0.1, tree complexity = 1 and number of trees = 100. An evaluation of the model prediction accuracy measured using the Area under Curve (AUC) over 25 iterations showed AUC = 0.89.

**Fig. 3:**
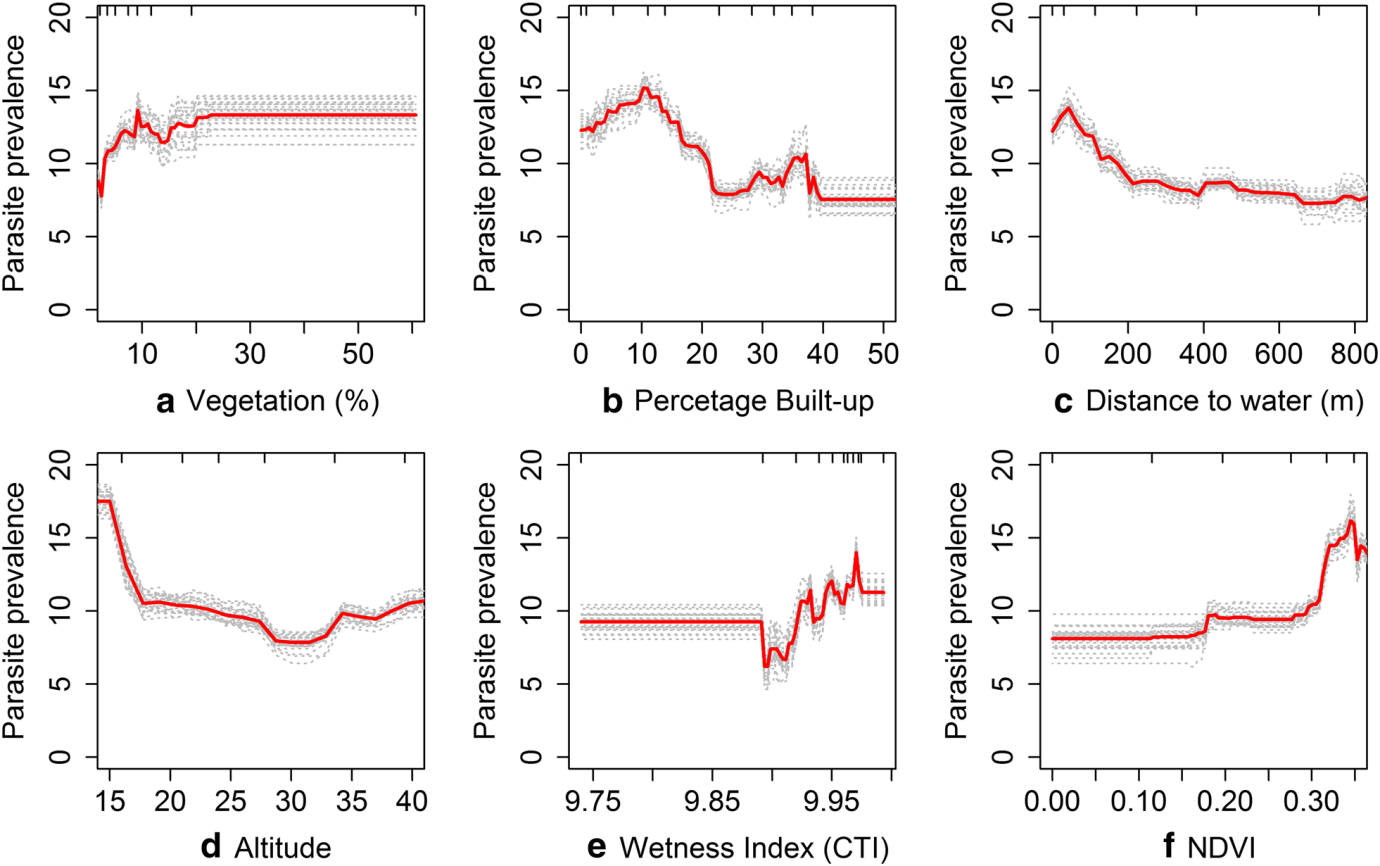
Partial dependence plots showing the effect of different environmental predictors on the *Pf*PR. *Note* effect of each environmental predictors after accounting for the average effect of other explanatory variables Results are shown for **a** vegetation with 1 km (%), **b** percentage built-up **c** Proximity to inland water, **d** altitude, **e** Wetness Index (CTI), **f** NDVI. Results of each of the 25 bootstrap runs are shown in *grey dashed lines* while average/mean plot is shown in *red line*

The relative contribution of significant predictor variables on the outcome (malaria positivity) is summarized in Table 2. Among the LC variables, the percentage of dense/riverine vegetation, built-up areas and proximity to water were found to be important predictors of *Pf*PR_2–10_. The percentage of dense/riverine vegetation within a 1 km radius was found to be the most important predictor of parasite prevalence with a relative contribution of close to 30 % (Table 2). Partial differential plots showing the effect of the environmental variables on the *Pf*PR are shown in Fig. 3. The risk of malaria infection was shown to increase as the percentage of dense vegetation increased. Urban land cover, measured using percentage built-up area, was ranked as the second most important predictor of parasite prevalence with a relative contribution of 27 % (Table 2). Partial responses indicated malaria infection risk decreased with increase in built-up land cover beyond 10 % of built-up areas. Proximity to inland water was found to be the third most important predictor of malaria infection risk, with an overall relative contribution of 9 % (Table 2). Communities closer to water bodies were found to be at a higher risk of infection than those living further away.

**Table 2:** Summary of the average contributions of the significant predictor variables using a Boosted Regression Trees (BRT) model

Variable	Average relative contribution (%)
Percentage dense/riverine vegetation	29.94
Percentage built-up	26.85
Distance to inland water (m)	8.98
Altitude	8.34
Wetness Index (CTI)	6.61
NDVI	5.44

BRT model developed with cross-validation over 25 bootstraps. Average relative contribution refers to the influence of each variable to the BRT model calculated as the proportion of times that a variable was selected for splitting, weighted by the squared improvement to the model as a result of each split [73]. This was then averaged over the 25 iterations of the BRT model run. This has added to the footnote of Table 2. Variables with zero influence or relative contribution <1 % were dropped from the analysis. Effect of other land cover classes was controlled for

Topographic variables were also found to influence parasite prevalence. Altitude derived from ASTER 30 m DEM was ranked 4th with 9 % relative contribution, with a trend toward malaria risk decreasing with increasing altitude. The topography Derived Wetness Index (CTI) was also shown to be associated with malaria infection risk, ranked as the fifth most important predictor and a relative contribution of approximately 7 % (Table 2). The partial differential plot indicates that wetter areas were associated with higher values of *Pf*PR_2–10_. NDVI showed similar trends with an increase in NDVI (above 0.15) associated with an increase in parasite prevalence. However, NDWI, temperature and precipitation performed poorly as predictors of malaria infection risk with <0.01 relative contribution and not included in the subsequent model.

**Fig. 4:**
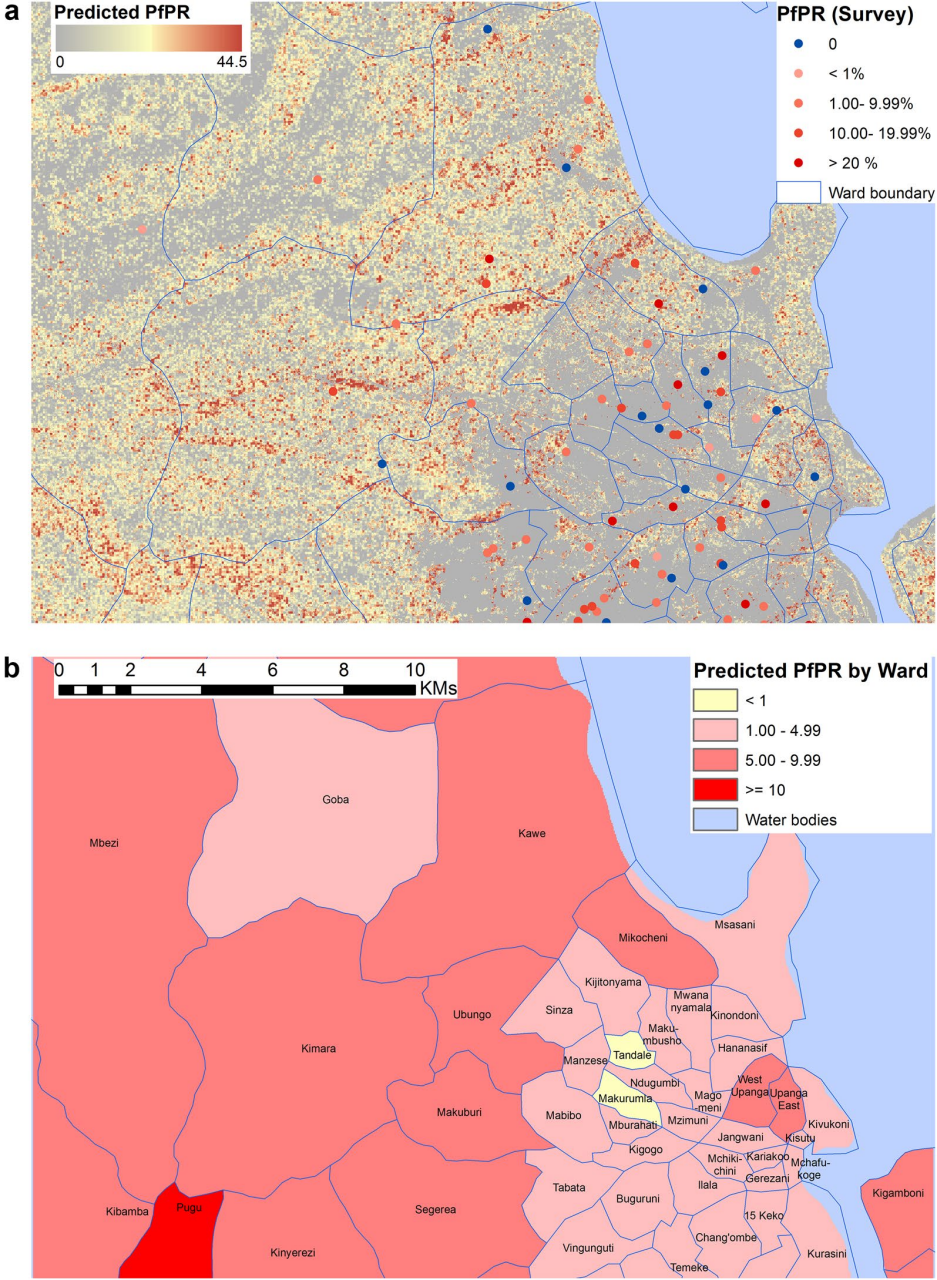
**a** Predicted *Pf*PR_2–10_ in Dar es Salaam against surveys conducted 2006–2014. **b** Summarised by Ward. *Note* **a** the distribution of community level surveys conducted between 2006 and 2014 (point data) is shown against the predicted *Pf*PR_2–10_ in Dar es Salaam in the background. In part (**b**), the predicted *Pf*PR_2–10_ predicted is summarized by wards, the lowest level of administration used by the municipal of Dar es Salaam

The optimal BRT model (Stage 3) was used to estimate parasite prevalence rates for each grid cell across Dar es Salaam with the predictions improved using bootstrapping techniques averaged over 25 iterations. The final ensemble prediction map identifies the spatial patterns of parasite prevalence across the city of Dar es Salaam (Fig. 4a). The spatial patterns of predicted parasite prevalence from the composite model suggest that the risk of malaria transmission increases away from the city centre. There is a higher risk of infection along water channels and close to dense vegetation and lower risks among the dense, built up areas of the city where vegetation is sparse (Fig. 4a). Transformation of these high resolution risk predictions to zonal estimates by administrative wards is shown in Fig. 4b. Predicted mean *Pf*PR_2–10_ ranged between 1 and 5 % in wards within central Dar es Salaam, with the lowest estimates, *Pf*PR_2–10_ less than 1 %, predicted in Makurumia and Tandale wards in the city centre (Fig. 4b). A slightly higher mean *Pf*PR_2–10_ of 6 % was predicted in Upanga ward, which borders mangrove swamps at the mouth of Msimbazi River. There was an increase in malaria prevalence in peri-urban wards as a result of increasing vegetation cover and decreasing built-up areas. The predicted mean *Pf*PR_2–10_ ranged between 5 and 10 % in the peri-urban areas. The highest mean *Pf*PR_2–10_ of above 10 % was predicted in Pugu ward in the outskirts of Dar es Salaam and close to the Pugu forest reserve (Fig. 4b).

## Discussion

Dar es Salaam, on the Tanzania coast, is characteristic of many rapidly growing, densely populated cities in Africa. Malaria transmission is generally considered lower in urban areas of Africa compared to neighbouring rural communities with an average entomological inoculation rates (EIR) of 18.8 infective bites per year estimated in urban areas compared to 126.3 in rural areas in a review of 33 independent surveys [7]; a similar trend was observed using 286 urban–rural pairs of parasite prevalence data [65]. However, the results of this study show that malaria risks do exist within the urban extents and that malaria risk within urban areas is not homogenous. In this study, we predicted heterogeneity in malaria risk using high resolution SPOT satellite image and ancillary environmental data without recourse to highly labour intensive ground mapping of risks. The variation in malaria risk within Dar es Salaam was shown to be influenced by varying environmental factors in different parts of the city with higher malaria risk associated with proximity to dense vegetation, inland water and wet/swampy areas while lower malaria risks were predicted in densely built-up areas. These results correspond to findings from mosquito vector abundance studies in Dakar where proximity to dense vegetation and large marshland areas was associated with increased mosquito densities and increased risk of malaria infection [8, 25, 66]. In Accra, Ghana, an increase in malaria cases was reported for people living within 1 km of urban agricultural activity [67] and in Ouagadougou, Burkina Faso, built up areas were significantly associated with declining malaria infection risks [24].

The findings of this study have implications for the recent efforts to model the intensity of malaria transmission across Africa through time [20–23]. These studies applied a single rule in all urban and peri-urban areas assuming malaria risk of infection was uniformly distributed within urban areas. However, the results of this study show that empirically measured risks across Dar es Salaam vary considerably and that this heterogeneity can be predicted based on the varying landscape within the city (Fig. 4a, b).

Remote sensing of the urban environment as used in this study offers some valuable information when aiming to identify areas within urban settlements in Africa where malaria continues to pose a significant problem. The predicted malaria risk map of Dar es Salaam, when reformatted to administrative areas used by the municipality (Fig. 4b), shows areas of high, moderate and low risk influenced by the distribution of predictor variables. These maps can serve as valuable resources for municipal councils aiming to shrink the extents of malaria across cities, target resources for vector control or intensify mosquito and disease surveillance. The semi-automated modelling process developed in this study can be updated with new data for monitoring and estimating trends in malaria risk over time. There is also potential to scale up malaria risk evaluation to other urban areas in Africa using the methods developed in this study which can easily be replicated to identify factors that influence heterogeneity in malaria risk patterns and detect vulnerable zones.

The current malaria control strategy in Dar es Salaam implemented by UMCP focuses on integrated malaria vector control based on ground-based mapping and surveillance of potential mosquito breeding sites. However, this has been reported as labour-intensive and expensive [15, 37] while the translation of entomological-based measures into disease outcomes, such as the prevalence of malaria infection, is not straightforward [15, 37]. Remote sensing of the urban environment as used in this study provides a faster and less labour intensive alternative for targeted and effective control application. We also explore the use of parasite prevalence which is simpler to measure in the field with standardized methods and has previously been used in urban settings [15, 24]. We used *Pf*PR measured at community level which allowed directly linkage to local environment characteristics when evaluating malaria risk factors. This is an advantage over infection estimates collected at health facilities that rarely include information on the community of residence of patients making it difficult to directly estimate the impact of environmental variables on local malaria risk. Further, community parasite prevalence surveys are obtained through active detection by screening populations irrespective of the presence symptoms of malaria unlike health facility level data that relies on symptomatic patients presenting for diagnosis and treatment. Finally, with community level parasite rates, populations at risk of malaria infection can be accurately estimated.

There are however some limitations when interpreting the results of this study. First, the temporal/seasonal trend in malaria risk could not be estimated in this study due to the unavailability of frequently collected parasite prevalence survey data that is well distributed across Dar es Salaam. We therefore used PR estimates aggregated over the period of study to predict a single risk map which does not account for change in environmental variables over the study period. Secondly, there were no details from the surveys included on whether infections were acquired locally, or whether an individual had travelled outside of his/her usual residence. The assumption made throughout this study has been that all infections were locally acquired from the point where someone was surveyed. However, studies suggest that some of malaria cases reported in urban areas are imported from travel to areas with high levels of malaria transmission and in the presence of proficient malaria vectors, increases malaria risk in urban areas [17, 18, 68, 69]. The inclusion of travel histories of children positive for malaria in future parasite prevalence surveys would therefore be important in overcoming this limitation and can improve the results of the study.

There are also some constraints in using geospatial datasets at varying scales as used in this study. Although high resolution data is necessary for accurate mapping of malaria risk, remote sensing datasets are rarely available at the high resolution needed and it is often necessary to combine datasets of varying resolution to estimate risk. All resampling methods introduce some level of spatial errors as they preserve the pattern recognized at coarser resolution without increasing the information content. Secondly, there are some constraints in identifying a limited number of land-cover classes when using very high resolution satellite imagery. To minimise this constraint, a two-step hybrid classification method was used to aggregate land cover classes with similar characteristics with numerous training samples distributed across the study area taken to account for spectral range within a land cover class.

In addition, by using parasite prevalence summarised at community level, variability in risk within the community is not accounted for and thus the estimated relationship with environmental variables is effective at the community level. There is potential to explore variability in predicted risk using higher resolution parasite prevalence data. A recent study in Mozambique showed that models matching national household-level malaria infection data to high resolution environmental datasets resulted in more precise prediction compared to models using lower resolution data of greater than 30 m [70]. There is need to explore the application of household level datasets with urban contexts in Africa. Lastly, in order to account for the true effect of time on environmental determinants of malaria, the environmental covariates used must be matched with the observed data on malaria transmission. However, the environmental datasets are rarely available at time points that correspond with the date of surveys as most are derived from long-term processed remotely sensed satellite imagery or modelled climatic data generated as synoptic estimates that do not represent a specific year [71, 72].

Finally, while the development of the malaria risk map does not require extensive ground survey work of environmental risk factors, there are a number of caveats to their wider applicability. First, the model development depends on high resolution satellite imagery that is not free to public health practitioners which limits their use in public health. Public health practitioners including NMCP could benefit from collaborations with donors that lower or subsidize the cost of high resolution satellite imagery. Second, the model requires that there are some survey data that enables a training of environmental data. This in itself is not a caveat, models unencumbered by data often do not reflect the complexities of disease under controlled and real life conditions. However, the implications are that there is a need to test the externality of findings presented here across of a range of urban settings in Africa where data do exist and their wider applications across Africa’s urban extents.

## Conclusion

With more than half of Africa’s population expected to live in urban settlements by 2030, the burden of malaria among urban populations in Africa will continue to rise with the increasing number of people at risk of infection. However, malaria intervention across Africa remains focused on rural, highly endemic communities with far fewer strategic policy directions for the control of malaria in rapidly growing African urban settlements. The complex and heterogeneous nature of urban malaria requires a better understanding of the spatial and temporal patterns of urban malaria risk in order to design effective urban malaria control programs targeted to specific zones. The semi-automated image classification and modelling process developed in this study can easily be replicated in other urban areas to identify environmental factors that influence heterogeneity in malaria risk patterns and detect zones of vulnerability. There is a definite need to expand research into the unique epidemiology of malaria transmission in urban areas for focal elimination and sustained control agendas.

## Additional file

10.1186/s12942-016-0051-y Descriptive Statistics of community level parasite prevalence surveys assembled in Dar es Salaam between 2006 and 2014.

## Abbreviations

ASTER: Advanced Spaceborne Thermal Emission and Reflection Radiometer
BRT: Boosted Regression Tree
CTI: Compound Topographic Index
DEM: Digital Elevation Model
DOS: Dark Object Subtraction model
GIS: Geographical Information Systems
INFORM: Information for Malaria Project
LC: land cover
LST: land surface temperatures
MODIS: Moderate Resolution Imaging Spectro-radiometer
NDVI: Normalized Difference Vegetation Index
NDWI: Normalized Difference Water Index
NMCP: National Malaria Control Programme
RF: random forest
RFE: rainfall estimates
*Pf*PR_2–10_: age standardised malaria parasite prevalence
SPOT: Système Pour l‘Observation de la Terre
UMCP: Urban Malaria Control Project
