# Supplementary material for: Mapping intra-urban malaria risk using high resolution satellite imagery: a case study of Dar es Salaam
Source: Int J Health Geogr. 2016 Jul 30;15:26. doi: 10.1186/s12942-016-0051-y (PMC4967308; doi:10.1186/s12942-016-0051-y)
Supplement: Supplementary file 1 — 10.1186/s12942-016-0051-y Descriptive Statistics of community level parasite prevalence surveys assembled in Dar es Salaam between 2006 and 2014. [file 12942_2016_51_MOESM1_ESM.docx]

**Additional file**

**Table S1**. Descriptive Statistics of community level parasite prevalence surveys assembled in Dar es Salaam between 2006 and 2014

| **District/Ward** | **Number of Cluster** | **Total Individuals examined** | **Start Month** | **Start Year** | **End Month** | **End Year** | **PfPR_2-10_ Range** | | **Mean *Pf*PR_2-10_** |
| --- | --- | --- | --- | --- | --- | --- | --- | --- | --- |
| **Ilala District** | **56** | **6622** | **1** | **2006** | **12** | **2014** | **0.00** | **36.41** | **9.69** |
| *Ukonga* | 7 | 434 | 1 | 2006 | 12 | 2014 | 0.00 | 15.41 | 4.19 |
| *Buguruni* | 7 | 1001 | 1 | 2006 | 8 | 2014 | 0.00 | 25.84 | 7.44 |
| *Jangwani* | 1 | 70 | 8 | 2010 | 8 | 2010 | 26.21 | 26.21 | 26.21 |
| *Upanga Mashariki* | 1 | 105 | 8 | 2014 | 8 | 2014 | 0.00 | 0.00 | 0.00 |
| *Kiwalani* | 1 | 113 | 8 | 2014 | 8 | 2014 | 0.00 | 0.00 | 0.00 |
| *Segerea* | 9 | 1153 | 1 | 2010 | 11 | 2014 | 0.00 | 35.07 | 13.23 |
| *Kitunda* | 2 | 206 | 8 | 2014 | 8 | 2014 | 0.00 | 1.51 | 0.76 |
| *Chanika* | 1 | 106 | 8 | 2014 | 8 | 2014 | 0.75 | 0.75 | 0.75 |
| *Tabata* | 1 | 17 | 1 | 2012 | 1 | 2012 | 6.59 | 6.59 | 6.59 |
| *Kinyerezi* | 1 | 112 | 4 | 2011 | 4 | 2011 | 32.76 | 32.76 | 32.76 |
| *llala* | 6 | 752 | 5 | 2006 | 7 | 2008 | 0.00 | 24.65 | 12.62 |
| *Mchikichini* | 3 | 780 | 5 | 2006 | 7 | 2008 | 8.69 | 11.01 | 9.96 |
| *Vingunguti* | 12 | 1523 | 1 | 2006 | 12 | 2014 | 0.00 | 22.89 | 8.63 |
| *Kipawa* | 4 | 250 | 2 | 2010 | 3 | 2012 | 4.31 | 36.41 | 16.26 |
|  |  |  |  |  |  |  |  |  |  |
| **Kinondoni District** | **55** | **7959** | **1** | **2006** | **11** | **2014** | **0.00** | **38.54** | **9.96** |
| *Magomeni* | 1 | 104 | 8 | 2014 | 8 | 2014 | 0.77 | 0.77 | 0.77 |
| *Mabibo* | 1 | 23 | 9 | 2008 | 9 | 2008 | 4.68 | 4.68 | 4.68 |
| *Manzese* | 1 | 15 | 3 | 2012 | 3 | 2012 | 7.47 | 7.47 | 7.47 |
| *Ubungo* | 2 | 480 | 4 | 2010 | 7 | 2011 | 5.18 | 17.88 | 11.53 |
| *Goba* | 1 | 94 | 8 | 2014 | 8 | 2014 | 1.71 | 1.71 | 1.71 |
| *Kawe* | 8 | 884 | 1 | 2007 | 11 | 2014 | 0.00 | 23.18 | 8.93 |
| *Kunduchi* | 1 | 24 | 9 | 2008 | 9 | 2008 | 0.00 | 0.00 | 0.00 |
| *Mbweni* | 1 | 98 | 8 | 2014 | 8 | 2014 | 0.00 | 0.00 | 0.00 |
| *Makuburi* | 1 | 17 | 9 | 2008 | 9 | 2008 | 0.00 | 0.00 | 0.00 |
| *Makumbusho* | 3 | 310 | 4 | 2010 | 8 | 2012 | 0.00 | 23.26 | 12.12 |
| *Kijitonyama* | 4 | 765 | 5 | 2010 | 11 | 2012 | 2.02 | 23.47 | 10.99 |
| *Kimara* | 2 | 214 | 7 | 2011 | 8 | 2014 | 0.00 | 12.54 | 6.27 |
| *Mikocheni* | 3 | 657 | 5 | 2007 | 8 | 2014 | 0.00 | 29.83 | 13.97 |
| *Mbezi* | 1 | 97 | 8 | 2014 | 8 | 2014 | 0.82 | 0.82 | 0.82 |
| *Hananasif* | 3 | 276 | 5 | 2010 | 8 | 2014 | 0.00 | 31.77 | 10.89 |
| *Ndugumbi* | 5 | 1524 | 1 | 2006 | 7 | 2008 | 0.00 | 18.34 | 11.87 |
| *Tandale* | 4 | 416 | 5 | 2007 | 11 | 2014 | 0.00 | 21.14 | 9.46 |
| *Mwananyamala* | 6 | 909 | 3 | 2006 | 9 | 2012 | 0.00 | 38.54 | 14.44 |
| *Msasani* | 1 | 99 | 8 | 2014 | 8 | 2014 | 1.62 | 1.62 | 1.62 |

**Table S1** (continued)

| **District/Ward** | | **Number of Cluster** | | **Total Individuals examined** | **Start Month** | | **Start Year** | **End Month** | **End Year** | **PfPR_2-10_ Range** | | | **Mean *Pf*PR_2-10_** |
| --- | --- | --- | --- | --- | --- | --- | --- | --- | --- | --- | --- | --- | --- |
| *Mzimuni* | | 3 | | 772 | 3 | | 2006 | 7 | 2012 | 0.00 | | 9.66 | 4.85 |
| *Kigogo* | | 3 | | 181 | 4 | | 2010 | 5 | 2011 | 20.56 | | 25.36 | 23.50 |
|  | |  | |  |  | |  |  |  |  | |  |  |
| **Temeke District** | | **58** | | **8887** | **1** | | **2006** | **12** | **2014** | **0.00** | | **38.83** | **11.06** |
| *Kigamboni* | | 1 | | 102 | 8 | | 2014 | 8 | 2014 | 0.00 | | 0.00 | 0.00 |
| *Charambe* | | 3 | | 258 | 1 | | 2011 | 11 | 2014 | 0.78 | | 10.67 | 6.73 |
| *Miburani* | | 4 | | 599 | 2 | | 2010 | 4 | 2012 | 3.34 | | 38.36 | 17.32 |
| *Temeke* | | 1 | | 19 | 12 | | 2011 | 12 | 2011 | 0.00 | | 0.00 | 0.00 |
| *Mtoni* | | 3 | | 941 | 5 | | 2006 | 8 | 2014 | 0.78 | | 18.60 | 9.94 |
| *Kurasini* | | 1 | | 564 | 5 | | 2007 | 5 | 2007 | 17.06 | | 17.06 | 17.06 |
| *Azimio* | | 2 | | 727 | 5 | | 2006 | 7 | 2007 | 12.30 | | 23.34 | 17.82 |
| *Tandika* | | 4 | | 268 | 1 | | 2010 | 9 | 2011 | 0.00 | | 33.82 | 13.05 |
| *Sandali* | | 8 | | 955 | 1 | | 2010 | 12 | 2014 | 0.00 | | 32.76 | 14.22 |
| *Vijibweni* | | 2 | | 120 | 4 | | 2011 | 8 | 2014 | 0.86 | | 38.83 | 19.85 |
| *Chang'ombe* | | 10 | | 2604 | 2 | | 2006 | 11 | 2014 | 1.57 | | 32.32 | 11.64 |
| *Mbagala Kuu* | | 3 | | 224 | 8 | | 2007 | 10 | 2014 | 0.00 | | 1.57 | 0.78 |
| *Makangarawe* | | 1 | | 19 | 12 | | 2011 | 12 | 2011 | 0.00 | | 0.00 | 0.00 |
| *Mji Mwema* | | 3 | | 179 | 5 | | 2006 | 6 | 2011 | 4.48 | | 14.01 | 7.71 |
| *Kisarawe II* | | 1 | | 102 | 8 | | 2014 | 8 | 2014 | 12.56 | | 12.56 | 12.56 |
| *Mbagala* | | 4 | | 407 | 1 | | 2010 | 7 | 2012 | 6.89 | | 17.71 | 11.86 |
| *Chamazi* | | 1 | | 43 | 8 | | 2014 | 8 | 2014 | 0.00 | | 0.00 | 0.00 |
| *Yombo Vituka* | | 6 | | 756 | 3 | | 2010 | 9 | 2014 | 1.22 | | 16.50 | 10.30 |
|  | |  | |  |  | |  |  |  |  | |  |  |
| ***Summary by District*** | | | | |  | |  |  |  |  | |  |  |
|  | | | **Ilala District** | | | **Kinondoni District** | | | | | **Temeke District** | | |
| Study Period | | | January 2006 - December 2014 | | | | | | | | | | |
| Number of Wards | | | 14 | | | 21 | | | | | 18 | | |
| Total Number of Clusters | | | 56 | | | 55 | | | | | 58 | | |
| Total Individuals examined | | | 6622 | | | 7959 | | | | | 8887 | | |
| Age range (Years) | | | 0-99 | | | 0-99 | | | | | 0-99 | | |
| *Pf*PR (Range) | | | 0 - 27.78 | | | 0 - 29.41 | | | | | 0 - 29.63 | | |
| *Pf*PR (Mean) | | | 7.94 | | | 8.06 | | | | | 8.96 | | |
| Testing Method | Microscopy | | 9 | | | 12 | | | | | 10 | | |
|  | RDT | | 47 | | | 43 | | | | | 48 | | |
